# Supplementary material for: Cardiac interventions in Wales: A comparison of benefits between NHS Wales specialties
Source: PLoS One. 2024 Feb 9;19(2):e0297049. doi: 10.1371/journal.pone.0297049 (PMC10857708; doi:10.1371/journal.pone.0297049)
Supplement: S1 Table — (DOCX) [file pone.0297049.s001.docx]

**Table S1. Significant associations to 5% level from unadjusted univariate analysis**

| Intervention | Category | | Covariate | | Odds Ratio | 95% CI (lower) | 95% CI (upper) | p-Value |
| --- | --- | --- | --- | --- | --- | --- | --- | --- |
| Cardiac Devices | Top5%-notTop5% | | WIMD_FIFTHCATREORDER - 4 | | 4.36 | 1.21 | 15.76 | 0.025 |
|  | Top5%-notTop5% | | 1.OUTLIER_PRE | | 4.57 | 1.88 | 11.09 | 0.001 |
|  | Top5%-notTop5% | | 1.Total_cost_pre_any | | 3.03 | 1.45 | 6.30 | 0.003 |
|  | TotalCost-NoCost (elective) | | 1.Total_cost_pre_any | | 2.66 | 1.81 | 3.91 | 0.000 |
|  | TotalCost-NoCost (emergency) | | COMORB24TO6CAT3 - 3 | | 2.25 | 1.11 | 4.57 | 0.024 |
|  | TotalCost-NoCost (emergency) | | 1.Total_cost_pre_any | | 4.24 | 2.50 | 7.19 | 0.000 |
|  | Top5%-ZeroCost | | WIMD_FIFTHCATREORDER - 4 | | 4.62 | 1.26 | 16.99 | 0.021 |
|  | Top5%-ZeroCost | | WIMD_FIFTHCATREORDER - 5 | | 3.96 | 1.05 | 14.90 | 0.042 |
|  | Top5%-ZeroCost | | 1.outlier_pre | | 6.34 | 2.39 | 16.84 | 0.000 |
|  | Top5%-ZeroCost | | 1.Total_cost_pre_any | | 4.67 | 2.21 | 9.83 | 0.000 |
|  | Top5%-notTop5% (elective) | | 1.outlier_pre | | 4.59 | 1.58 | 13.35 | 0.005 |
|  | Top5%-notTop5% (emergency) | | 1.Total_cost_pre_any | | 6.64 | 2.08 | 21.20 | 0.001 |
| CABG | TotalCost-NoCost | | 3+ comorbidities | | 1.58 | 1.06 | 2.34 | 0.024 |
|  | TotalCost-NoCost | | 1.outlier_pre | | 1.90 | 1.16 | 3.13 | 0.011 |
|  | TotalCost-NoCost | | 1.Total_cost_pre_any | | 1.76 | 1.34 | 2.31 | 0.000 |
|  | TotalCost-NoCost (elective) | | 3+ comorbidities | | 1.78 | 1.00 | 3.15 | 0.049 |
|  | TotalCost-NoCost (elective) | | 1.outlier_pre | | 2.23 | 1.24 | 4.00 | 0.007 |
|  | TotalCost-NoCost (elective) | | 1.Total_cost_pre_any | | 2.25 | 1.52 | 3.32 | 0.000 |
|  | TotalCost-NoCost (emergency) | | 1.Total_cost_pre_any | | 1.97 | 1.20 | 3.23 | 0.007 |
|  | Top5%-notTop5% | | 3+ comorbidities | | 1.99 | 1.08 | 3.68 | 0.028 |
|  | Top5%-notTop5% | | 1.Total_cost_pre_any | | 1.67 | 1.05 | 2.66 | 0.030 |
|  | Top5%-notTop5% (elective) | | 2 comorbidities | | 2.93 | 1.19 | 7.22 | 0.020 |
|  | Top5%-notTop5% (elective) | | 1.outlier_pre | | 3.02 | 1.26 | 7.22 | 0.013 |
|  | Top5%-notTop5% (elective) | | 1.Total_cost_pre_any | | 2.62 | 1.29 | 5.35 | 0.008 |
|  | Top5%-notTop5% (emergency) | | 3+ comorbidities | | 2.50 | 1.16 | 5.42 | 0.020 |
|  | Top5%-ZeroCost | | 3+ comorbidities | | 2.08 | 1.12 | 3.86 | 0.020 |
|  | Top5%-ZeroCost | | 1.outlier_pre | | 2.34 | 1.08 | 5.06 | 0.032 |
|  | Top5%-ZeroCost | | 1.Total_cost_pre_any | | 1.81 | 1.13 | 2.88 | 0.013 |
| EP standard | TotalCost-NoCost | | 1.Total_cost_pre_any | | 2.17 | 1.43 | 3.31 | 0.000 |
|  | Top5%-notTop5% (elective) | | 2.Under 65 | | 0.37 | 0.14 | 0.98 | 0.045 |
| EP complex | TotalCost-NoCost | | AnyCostPre | | 2.27 | 1.35 | 3.83 | 0.002 |
|  | TotalCost-NoCost (elective) | | AnyCostPre | | 2.42 | 1.42 | 4.13 | 0.001 |
| EP study | TotalCost-NoCost | | AnyCostPre | | 2.14 | 1.01 | 4.54 | 0.048 |
| TAVI | TotalCost-NoCost | | 4: 2^nd^ most deprived | | 0.09 | 0.01 | 0.77 | 0.028 |
|  | Top5%-notTop5% | | OutlierPre | | 21.37 | 3.54 | 129.13 | 0.001 |
|  | Top5%-ZeroCost | | OutlierPre | | 19.13 | 3.16 | 115.66 | 0.001 |
| Valve | TotalCost-NoCost | | 5: Most Deprived | | 1.51 | 1.01 | 2.26 | 0.042 |
|  | TotalCost-NoCost | | 3+ comorbidities | | 1.89 | 1.22 | 2.92 | 0.004 |
|  | TotalCost-NoCost | | 1.outlier_pre | | 2.52 | 1.37 | 4.63 | 0.003 |
|  | TotalCost-NoCost | | 1.Total_cost_pre_any | | 2.57 | 1.96 | 3.37 | 0.000 |
|  | TotalCost-NoCost | | 1.rural_cat | | 0.68 | 0.51 | 0.91 | 0.008 |
|  | TotalCost-NoCost (elective) | | 3+ comorbidities | | 1.75 | 1.05 | 2.93 | 0.032 |
|  | TotalCost-NoCost (elective) | | 1.Total_cost_pre_any | | 2.16 | 1.58 | 2.97 | 0.000 |
|  | TotalCost-NoCost (elective) | | 1.rural_cat | | 0.72 | 0.52 | 0.99 | 0.042 |
|  | TotalCost-NoCost (emergency) | | 3+ comorbidities | | 2.40 | 1.02 | 5.65 | 0.045 |
|  | TotalCost-NoCost (emergency) | | 1.outlier_pre | | 3.65 | 1.22 | 10.91 | 0.021 |
|  | TotalCost-NoCost (emergency) | | 1.Total_cost_pre_any | | 5.91 | 3.09 | 11.30 | 0.000 |
|  | Top5%-notTop5% | | 2.gndr_cat | | 0.54 | 0.29 | 0.99 | 0.047 |
|  | Top5%-notTop5% | | 3+ comorbidities | | 2.80 | 1.26 | 6.22 | 0.012 |
|  | Top5%-notTop5% | | Unknown | | 6.74 | 1.78 | 25.56 | 0.005 |
|  | Top5%-notTop5% | | 1.outlier_pre | | 3.12 | 1.25 | 7.79 | 0.015 |
|  | Top5%-notTop5% (elective) | | 2.gndr_cat | | 0.40 | 0.18 | 0.88 | 0.023 |
|  | Top5%-notTop5% (elective) | | 3+ comorbidities | | 3.05 | 1.09 | 8.54 | 0.034 |
|  | Top5%-notTop5% (emergency) | | 1.outlier_pre | | 5.55 | 1.53 | 20.04 | 0.009 |
|  | Top5%-notTop5% (emergency) | | 1.Total_cost_pre_any | | 4.02 | 1.36 | 11.84 | 0.012 |
|  | Top5%-ZeroCost | | 3+ comorbidities | | 3.52 | 1.54 | 8.05 | 0.003 |
|  | Top5%-ZeroCost | | 3.Unknown | | 6.29 | 1.54 | 25.68 | 0.010 |
|  | Top5%-ZeroCost | | 1.outlier_pre | | 4.63 | 1.73 | 12.42 | 0.002 |
|  | Top5%-ZeroCost | | 1.Total_cost_pre_any | | 2.07 | 1.11 | 3.86 | 0.021 |
| PCI | TotalCost-NoCost | | 5: Most Deprived | | 1.43 | 1.17 | 1.74 | 0.000 |
|  | TotalCost-NoCost | | 1 comorbidity | | 1.42 | 1.21 | 1.66 | 0.000 |
|  | TotalCost-NoCost | | 2.Emergency | | 0.66 | 0.57 | 0.75 | 0.000 |
|  | TotalCost-NoCost | | 3.Unknown | | 0.40 | 0.23 | 0.71 | 0.002 |
|  | TotalCost-NoCost | | OutlierPre | | 5.07 | 4.00 | 6.42 | 0.000 |
|  | TotalCost-NoCost | | AnyCostPre | | 7.35 | 6.27 | 8.60 | 0.000 |
|  | TotalCost-NoCost | | Rural | | 0.74 | 0.64 | 0.85 | 0.000 |
|  | TotalCost-NoCost (elective) | | OutlierPre | | 2.28 | 1.63 | 3.20 | 0.000 |
|  | TotalCost-NoCost (elective) | | AnyCostPre | | 3.39 | 2.66 | 4.32 | 0.000 |
|  | TotalCost-NoCost (elective) | | Rural | | 0.71 | 0.55 | 0.93 | 0.011 |
|  | TotalCost-NoCost (emergency) | | 5: Most Deprived | | 1.56 | 1.22 | 1.99 | 0.000 |
|  | TotalCost-NoCost (emergency) | | 1 comorbidity | | 1.40 | 1.15 | 1.70 | 0.001 |
|  | TotalCost-NoCost (emergency) | | 3+ comorbidities | | 1.40 | 1.06 | 1.87 | 0.020 |
|  | TotalCost-NoCost (emergency) | | OutlierPre | | 10.25 | 7.11 | 14.78 | 0.000 |
|  | TotalCost-NoCost (emergency) | | AnyCostPre | | 14.29 | 11.29 | 18.08 | 0.000 |
|  | TotalCost-NoCost (emergency) | | Rural | | 0.77 | 0.65 | 0.91 | 0.002 |
|  | Top5%-notTop5% | | 3: middle deprivation level | | 1.57 | 1.09 | 2.26 | 0.015 |
|  | Top5%-notTop5% | | 1 comorbidity | | 1.96 | 1.48 | 2.60 | 0.000 |
|  | Top5%-notTop5% | | 2 comorbidities | | 2.01 | 1.33 | 3.02 | 0.001 |
|  | Top5%-notTop5% | | 3+ comorbidities | | 2.79 | 1.95 | 4.00 | 0.000 |
|  | Top5%-notTop5% | | 2.Emergency | | 0.78 | 0.60 | 1.00 | 0.049 |
|  | Top5%-notTop5% | | OutlierPre | | 7.41 | 5.50 | 9.97 | 0.000 |
|  | Top5%-notTop5% | | AnyCostPre | | 6.42 | 5.04 | 8.16 | 0.000 |
|  | Top5%-notTop5% (elective) | | 2.Under 65 | | 0.57 | 0.33 | 0.99 | 0.048 |
|  | Top5%-notTop5% (elective) | | 3+ comorbidities | | 2.59 | 1.37 | 4.87 | 0.003 |
|  | Top5%-notTop5% (elective) | | OutlierPre | | 5.77 | 3.63 | 9.18 | 0.000 |
|  | Top5%-notTop5% (elective) | | AnyCostPre | | 5.24 | 3.36 | 8.15 | 0.000 |
|  | Top5%-notTop5% (emergency) | | 3: middle deprivation level | | 1.70 | 1.08 | 2.67 | 0.021 |
|  | Top5%-notTop5% (emergency) | | 1 comorbidity | | 2.22 | 1.58 | 3.10 | 0.000 |
|  | Top5%-notTop5% (emergency) | | 2 comorbidities | | 2.19 | 1.31 | 3.68 | 0.003 |
|  | Top5%-notTop5% (emergency) | | 3+ comorbidities | | 2.79 | 1.80 | 4.34 | 0.000 |
|  | Top5%-notTop5% (emergency) | | OutlierPre | | 9.07 | 6.06 | 13.58 | 0.000 |
|  | Top5%-notTop5% (emergency) | | AnyCostPre | | 7.95 | 5.87 | 10.77 | 0.000 |
|  | Top5%-ZeroCost | | 3: middle deprivation level | | 1.60 | 1.11 | 2.30 | 0.012 |
|  | Top5%-ZeroCost | | 5: Most Deprived | | 1.46 | 1.01 | 2.12 | 0.047 |
|  | Top5%-ZeroCost | | 1 comorbidity | | 2.04 | 1.54 | 2.71 | 0.000 |
|  | Top5%-ZeroCost | | 2 comorbidities | | 2.03 | 1.34 | 3.06 | 0.001 |
|  | Top5%-ZeroCost | | 3+ comorbidities | | 2.73 | 1.90 | 3.93 | 0.000 |
|  | Top5%-ZeroCost | | 2.Emergency | | 0.72 | 0.55 | 0.92 | 0.010 |
|  | Top5%-ZeroCost | | OutlierPre | | 10.45 | 7.62 | 14.33 | 0.000 |
|  | Top5%-ZeroCost | | AnyCostPre | | 10.76 | 8.37 | 13.84 | 0.000 |
|  | | | | | | | | |
| Sex | | Females (Baseline); | | Males | | | | |
| Age Group (yrs) | | 75-84 (Baseline) | | 2.Under 65; 3. 65-74; 4. 85+ | | | | |
| Admission type | | 1.Elective (Baseline) | | 2.Emergency; 3 Unknown | | | | |
| Deprivation quintiles | | 1. Least Deprived (Baseline) | | 2: 2^nd^ least deprived; 3: middle deprivation level; 4: 2^nd^ most deprived; 5: Most Deprived | | | | |
| OutlierPre | | No (Baseline); | | Yes | | | | |
| AnyCostPre | | No (Baseline); | | Yes | | | | |
| Comorbidities | | 0: No comorbidities (baseline) | | 1: 1 comorbidity; 2: 2 comorbidities; 3: 3+ comorbidities | | | | |
| Rurality | | No-Urban (Baseline); | | Yes-Rural | | | | |
