# Supplementary material for: Cardiac interventions in Wales: A comparison of benefits between NHS Wales specialties
Source: PLoS One. 2024 Feb 9;19(2):e0297049. doi: 10.1371/journal.pone.0297049 (PMC10857708; doi:10.1371/journal.pone.0297049)
Supplement: S2 Table — (DOCX) [file pone.0297049.s002.docx]

**Table S2. Significant associations to 5% level from multivariate analysis**

| Intervention | Category | | Covariate | | Odds Ratio | 95% CI (lower) | 95% CI (upper) | p-Value |
| --- | --- | --- | --- | --- | --- | --- | --- | --- |
| Cardiac Devices | TotalCost-NoCost | | 3+ comorbidities | | 0.52 | 0.32 | 0.84 | 0.008 |
|  | TotalCost-NoCost | | 1.Total_cost_pre_any | | 3.86 | 2.66 | 5.59 | 0.000 |
|  | TotalCost-NoCost (elective) | | 2.Under 65 | | 0.52 | 0.28 | 0.94 | 0.030 |
|  | TotalCost-NoCost (elective) | | 3+ comorbidities | | 0.44 | 0.24 | 0.80 | 0.007 |
|  | TotalCost-NoCost (elective) | | 1.Total_cost_pre_any | | 3.24 | 2.05 | 5.15 | 0.000 |
|  | TotalCost-NoCost (emergency) | | 2 comorbidities | | 0.30 | 0.11 | 0.81 | 0.017 |
|  | TotalCost-NoCost (emergency) | | 1.Total_cost_pre_any | | 5.75 | 2.87 | 11.52 | 0.000 |
|  | Top5%-notTop5% | | 1.outlier_pre | | 2.81 | 1.04 | 7.59 | 0.041 |
|  | Top5%-notTop5% | | 1.Total_cost_pre_any | | 3.98 | 1.62 | 9.77 | 0.003 |
|  | Top5%-notTop5% (elective) | | 1.outlier_pre | | 3.53 | 1.03 | 12.13 | 0.045 |
|  | Top5%-notTop5% (emergency) | | 1.Total_cost_pre_any | | 7.45 | 1.84 | 30.21 | 0.005 |
|  | Top5%-ZeroCost | | 1.Total_cost_pre_any | | 6.48 | 2.60 | 16.20 | 0.000 |
| CABG | TotalCost-NoCost | | 3+ comorbidities | | 1.47 | 1.10 | 1.98 | 0.009 |
|  | TotalCost-NoCost | | 1.Total_cost_pre_any | | 1.95 | 1.39 | 2.73 | 0.000 |
|  | TotalCost-NoCost (elective) | | 1.Total_cost_pre_any | | 2.09 | 1.36 | 3.22 | 0.001 |
|  | TotalCost-NoCost (emergency) | | 1.Total_cost_pre_any | | 2.13 | 1.20 | 3.76 | 0.010 |
|  | Top5%-notTop5% (emergency) | | 3+ comorbidities | | 2.49 | 1.09 | 5.68 | 0.031 |
|  | Top5%-ZeroCost | | 1.Total_cost_pre_any | | 1.85 | 1.03 | 3.33 | 0.040 |
| EP standard | TotalCost-NoCost | | 1.Total_cost_pre_any | | 2.11 | 1.35 | 3.30 | 0.001 |
| EP complex | TotalCost-NoCost | | 1.Total_cost_pre_any | | 2.58 | 1.46 | 4.57 | 0.001 |
|  | TotalCost-NoCost (elective) | | 1.Total_cost_pre_any | | 2.97 | 1.65 | 5.33 | 0.000 |
|  | Top5%-notTop5% | | 2 comorbidities | | 7.61 | 1.13 | 51.31 | 0.037 |
|  | Top5%-notTop5% (elective) | | 2 comorbidities | | 7.61 | 1.13 | 51.31 | 0.037 |
| EP study | (none) | | (none) | | - | - | - | - |
| TAVI | TotalCost-NoCost | | 4: 2^nd^ most deprived | | 0.05 | 0.00 | 0.54 | 0.014 |
| Valve | TotalCost-NoCost | | AnyCostPre | | 2.56 | 1.89 | 3.48 | 0.000 |
|  | TotalCost-NoCost (elective) | | AnyCostPre | | 2.00 | 1.43 | 2.79 | 0.000 |
|  | TotalCost-NoCost (emergency) | | AnyCostPre | | 10.03 | 4.07 | 24.68 | 0.000 |
|  | Top5%-notTop5% | | Sex=Male | | 0.52 | 0.28 | 0.97 | 0.039 |
|  | Top5%-notTop5% | | AdmissionType=Unknown | | 7.57 | 1.80 | 31.91 | 0.006 |
|  | Top5%-notTop5% (elective) | | Sex=Male | | 0.40 | 0.18 | 0.90 | 0.027 |
|  | Top5%-ZeroCost | | AdmissionType=Unknown | | 10.94 | 2.33 | 51.26 | 0.002 |
|  | Top5%-ZeroCost | | AnyCostPre | | 2.61 | 1.21 | 5.60 | 0.014 |
| PCI | TotalCost-NoCost | | 2.Under 65 | | 1.23 | 1.01 | 1.48 | 0.035 |
|  | TotalCost-NoCost | | AnyCostPre | | 7.55 | 6.21 | 9.18 | 0.000 |
|  | TotalCost-NoCost | | Rural | | 0.81 | 0.70 | 0.95 | 0.009 |
|  | TotalCost-NoCost (elective) | | 2 comorbidities | | 0.64 | 0.41 | 0.99 | 0.045 |
|  | TotalCost-NoCost (elective) | | AnyCostPre | | 3.72 | 2.78 | 4.98 | 0.000 |
|  | TotalCost-NoCost (elective) | | Rural | | 0.74 | 0.56 | 0.98 | 0.033 |
|  | TotalCost-NoCost (emergency) | | 5: Most Deprived | | 1.34 | 1.03 | 1.76 | 0.032 |
|  | TotalCost-NoCost (emergency) | | AnyCostPre | | 13.33 | 10.13 | 17.55 | 0.000 |
|  | Top5%-notTop5% | | 1 comorbidity | | 1.44 | 1.07 | 1.94 | 0.016 |
|  | Top5%-notTop5% | | 3+ comorbidities | | 1.52 | 1.01 | 2.27 | 0.044 |
|  | Top5%-notTop5% | | 2.Emergency | | 1.40 | 1.06 | 1.86 | 0.019 |
|  | Top5%-notTop5% | | OutlierPre | | 1.92 | 1.31 | 2.80 | 0.001 |
|  | Top5%-notTop5% | | AnyCostPre | | 4.89 | 3.59 | 6.67 | 0.000 |
|  | Top5%-notTop5% (elective) | | OutlierPre | | 2.26 | 1.25 | 4.05 | 0.007 |
|  | Top5%-notTop5% (elective) | | AnyCostPre | | 3.81 | 2.21 | 6.56 | 0.000 |
|  | Top5%-notTop5% (emergency) | | 3: middle deprivation level | | 1.63 | 1.01 | 2.61 | 0.044 |
|  | Top5%-notTop5% (emergency) | | 1 comorbidity | | 1.78 | 1.25 | 2.54 | 0.001 |
|  | Top5%-notTop5% (emergency) | | OutlierPre | | 1.75 | 1.05 | 2.94 | 0.033 |
|  | Top5%-notTop5% (emergency) | | AnyCostPre | | 5.82 | 4.00 | 8.47 | 0.000 |
|  | Top5%-ZeroCost | | 1 comorbidity | | 1.42 | 1.04 | 1.93 | 0.028 |
|  | Top5%-ZeroCost | | 2.Emergency | | 1.73 | 1.27 | 2.35 | 0.000 |
|  | Top5%-ZeroCost | | OutlierPre | | 1.68 | 1.11 | 2.54 | 0.013 |
|  | Top5%-ZeroCost | | AnyCostPre | | 9.84 | 7.01 | 13.80 | 0.000 |
|  | | | | | | | | |
| Sex | | Females (Baseline); | | Males | | | | |
| Age Group (yrs) | | 75-84 (Baseline) | | 2.Under 65; 3. 65-74; 4. 85+ | | | | |
| Admission type | | 1.Elective (Baseline) | | 2.Emergency; 3 Unknown | | | | |
| Deprivation quintiles | | 1. Least Deprived (Baseline) | | 2: 2^nd^ least deprived; 3: middle deprivation level; 4: 2^nd^ most deprived; 5: Most Deprived | | | | |
| OutlierPre | | No (Baseline); | | Yes | | | | |
| AnyCostPre | | No (Baseline); | | Yes | | | | |
| Comorbidities | | 0: No comorbidities (baseline) | | 1: 1 comorbidity; 2: 2 comorbidities; 3: 3+ comorbidities | | | | |
| Rurality | | No-Urban (Baseline); | | Yes-Rural | | | | |
