# Supplementary material for: Cardiac interventions in Wales: A comparison of benefits between NHS Wales specialties
Source: PLoS One. 2024 Feb 9;19(2):e0297049. doi: 10.1371/journal.pone.0297049 (PMC10857708; doi:10.1371/journal.pone.0297049)
Supplement: S3 Table — (DOCX) [file pone.0297049.s003.docx]

**Table A1: Codes used to define interventions**

| Intervention | Diagnostic codes | Code type |
| --- | --- | --- |
| Cardiac Devices | K591, K592, K594, K596 | OPCS |
| CABG | ED22A, ED22B, ED22C, ED23A, ED23B, ED23C, ED26A, ED26B, ED26C, ED27A, ED27B, ED27C, ED28A, ED28B, ED28C | HRG |
| EP standard | EY31A, EY31B | HRG |
| EP complex | EY30A, EY30B | HRG |
| EP study | EY32A, EY32B | HRG |
| TAVI | EY21A, EY21B | HRG |
| VALVE | ED20A, ED20B, ED21A, ED24A, ED24B, ED24C, ED25A, ED25B, ED25C | HRG |
| PCI | EY40A, EY40B, EY40C, EY40D, EY41A, EY41B, EY41C, EY41D | HRG |
