# Supplementary material for: Cardiac interventions in Wales: A comparison of benefits between NHS Wales specialties
Source: PLoS One. 2024 Feb 9;19(2):e0297049. doi: 10.1371/journal.pone.0297049 (PMC10857708; doi:10.1371/journal.pone.0297049)
Supplement: S4 Table — (DOCX) [file pone.0297049.s004.docx]

**Table A2: Codes used to define conditions**

| **Condition** | **Diagnostic codes** | **Code type** |
| --- | --- | --- |
| Atrial fibrillation | G573. | Read |
|  | I480, I481, I482, I483, I484, I489, I48X | ICD-10 |
| CAMHS/Mental health | E10..% , E110.% , E111.% , E1124 , E1134 , E114.-E117z , E11y.% (excluding E11y2) , E11z. , E11z0 , E11zz , E12..% , E13..% (excluding E135.) , E2122 , Eu2..% , Eu30.% , Eu31.% , Eu323 , Eu328 , Eu333 , Eu32A , Eu329, d6...% | Read |
|  | F000, F001, F002, F009, F010, F011, F012, F013, F018, F019, F020, F021, F022, F023, F024, F028, F03X, F04X, F050, F051, F058, F059, F060, F061, F062, F063, F064, F065, F066, F067, F068, F069, F070, F071, F072, F078, F079, F09X, F104, F106, F108, F109, F114, F116, F118, F119, F124, F126, F128, F129, F134, F136, F138, F139, F144, F146, F148, F149, F154, F156, F158, F159, F164, F166, F168, F169, F174, F176, F178, F179, F184, F186, F188, F189, F194, F196, F198, F199, F320, F321, F322, F328, F329, F330, F331, F332, F334, F338, F339, F340, F341, F348, F349, F380, F381, F388, F39X, F400, F401, F402, F408, F409, F410, F411, F412, F413, F418, F419, F420, F421, F422, F428, F429, F430, F431, F432, F438, F439, F440, F441, F442, F443, F444, F445, F446, F447, F448, F449, F450, F451, F452, F453, F454, F458, F459, F480, F481, F488, F489, F500, F501, F502, F503, F504, F505, F508, F509, F510, F511, F512, F513, F514, F515, F518, F519, F520, F521, F522, F523, F524, F525, F526, F527, F528, F529, F530, F531, F538, F539, F54X, F55X, F59X, F600, F601, F602, F603, F604, F605, F606, F607, F608, F609, F61X, F620, F621, F628, F629, F630, F631, F632, F633, F638, F639, F640, F641, F642, F648, F649, F650, F651, F652, F653, F654, F655, F656, F658, F659, F660, F661, F662, F668, F669, F680, F681, F688, F69X, F910, F911, F912, F913, F918, F919, F920, F928, F929, F930, F931, F932, F933, F938, F939, F940, F941, F942, F948, F949, F950, F951, F952, F958, F959, F980, F981, F982, F983, F984, F985, F986, F988, F989, F99X, G300, G301, G308, G309, Z032, Z046, Z093, Z133, Z504, Z543, Z550, Z551, Z552, Z553, Z554, Z558, Z559, Z560, Z561, Z562, Z563, Z564, Z565, Z566, Z567, Z590, Z591, Z592, Z593, Z594, Z595, Z596, Z597, Z598, Z599, Z600, Z601, Z602, Z603, Z604, Z605, Z608, Z609, Z610, Z611, Z612, Z613, Z614, Z615, Z616, Z617, Z618, Z619, Z620, Z621, Z622, Z623, Z624, Z625, Z626, Z628, Z629, Z630, Z631, Z632, Z633, Z634, Z635, Z636, Z637, Z638, Z639, Z640, Z641, Z642, Z643, Z644, Z650, Z651, Z652, Z653, Z654, Z655, Z658, Z659, Z700, Z701, Z702, Z703, Z708, Z709, Z718, Z719, Z726, Z730, Z731, Z732, Z733, Z765, Z818, Z865, Z914, Z915 | ICD-10 |
| Cancer | B0...-B32z. , B34..-B6z0. (excluding B677.) , Byu..- Byu41 , Byu5.-ByuE0 , K1323 , K01w1 , 68W24 , C184. | Read |
|  | C000, C001, C002, C003, C004, C005, C006, C008, C009, C01X, C020, C021, C022, C023, C024, C028, C029, C030, C031, C039, C040, C041, C048, C049, C050, C051, C052, C058, C059, C060, C061, C062, C068, C069, C07X, C080, C081, C088, C089, C090, C091, C098, C099, C100, C101, C102, C103, C104, C108, C109, C110, C111, C112, C113, C118, C119, C12X, C130, C131, C132, C138, C139, C140, C142, C148, C150, C151, C152, C153, C154, C155, C158, C159, C160, C161, C162, C163, C164, C165, C166, C168, C169, C170, C171, C172, C173, C178, C179, C180, C181, C182, C183, C184, C185, C186, C187, C188, C189, C19X, C20X, C210, C211, C212, C218, C220, C221, C222, C223, C224, C227, C229, C23X, C240, C241, C248, C249, C250, C251, C252, C253, C254, C257, C258, C259, C260, C261, C268, C269, C300, C301, C310, C311, C312, C313, C318, C319, C320, C321, C322, C323, C328, C329, C33X, C340, C341, C342, C343, C348, C349, C37X, C380, C381, C382, C383, C384, C388, C390, C398, C399, C400, C401, C402, C403, C408, C409, C410, C411, C412, C413, C414, C418, C419, C430, C431, C432, C433, C434, C435, C436, C437, C438, C439, C440, C441, C442, C443, C444, C445, C446, C447, C448, C449, C450, C451, C452, C457, C459, C460, C461, C462, C463, C467, C468, C469, C470, C471, C472, C473, C474, C475, C476, C478, C479, C480, C481, C482, C488, C490, C491, C492, C493, C494, C495, C496, C498, C499, C500, C501, C502, C503, C504, C505, C506, C508, C509, C510, C511, C512, C518, C519, C52X, C530, C531, C538, C539, C540, C541, C542, C543, C548, C549, C55X, C56X, C570, C571, C572, C573, C574, C577, C578, C579, C58X, C600, C601, C602, C608, C609, C61X, C620, C621, C629, C630, C631, C632, C637, C638, C639, C64X, C65X, C66X, C670, C671, C672, C673, C674, C675, C676, C677, C678, C679, C680, C681, C688, C689, C690, C691, C692, C693, C694, C695, C696, C698, C699, C700, C701, C709, C710, C711, C712, C713, C714, C715, C716, C717, C718, C719, C720, C721, C722, C723, C724, C725, C728, C729, C73X, C740, C741, C749, C750, C751, C752, C753, C754, C755, C758, C759, C760, C761, C762, C763, C764, C765, C767, C768, C770, C771, C772, C773, C774, C775, C778, C779, C780, C781, C782, C783, C784, C785, C786, C787, C788, C790, C791, C792, C793, C794, C795, C796, C797, C798, C80X, C810, C811, C812, C813, C817, C819, C820, C821, C822, C827, C829, C830, C831, C832, C833, C834, C835, C836, C837, C838, C839, C840, C841, C842, C843, C844, C845, C848, C850, C851, C852, C857, C859, C880, C881, C882, C883, C887, C889, C900, C901, C902, C910, C911, C912, C913, C914, C915, C917, C919, C920, C921, C922, C923, C924, C925, C927, C929, C930, C931, C932, C937, C939, C940, C941, C942, C943, C944, C945, C947, C950, C951, C952, C957, C959, C960, C961, C962, C963, C967, C969, C97X, D000, D001, D002, D010, D011, D012, D013, D014, D015, D017, D019, D020, D021, D022, D023, D024, D030, D031, D032, D033, D034, D035, D036, D037, D038, D039, D040, D041, D042, D043, D044, D045, D046, D047, D048, D049, D050, D051, D057, D059, D060, D061, D067, D069, D070, D071, D072, D073, D074, D075, D076, D090, D091, D092, D093, D097, D099, D100, D101, D102, D103, D104, D105, D106, D107, D109, D110, D117, D119, D120, D121, D122, D123, D124, D125, D126, D127, D128, D129, D130, D131, D132, D133, D134, D135, D136, D137, D139, D140, D141, D142, D143, D144, D150, D151, D152, D157, D159, D160, D161, D162, D163, D164, D165, D166, D167, D168, D169, D170, D171, D172, D173, D174, D175, D176, D177, D179, D180, D181, D190, D191, D197, D199, D200, D201, D210, D211, D212, D213, D214, D215, D216, D219, D220, D221, D222, D223, D224, D225, D226, D227, D229, D230, D231, D232, D233, D234, D235, D236, D237, D239, D24X, D250, D251, D252, D259, D260, D261, D267, D269, D27X, D280, D281, D282, D287, D289, D290, D291, D292, D293, D294, D297, D299, D300, D301, D302, D303, D304, D307, D309, D310, D311, D312, D313, D314, D315, D316, D319, D320, D321, D329, D330, D331, D332, D333, D334, D337, D339, D34X, D350, D351, D352, D353, D354, D355, D356, D357, D358, D359, D360, D361, D367, D369, D370, D371, D372, D373, D374, D375, D376, D377, D379, D380, D381, D382, D383, D384, D385, D386, D390, D391, D392, D397, D399, D400, D401, D407, D409, D410, D411, D412, D413, D414, D417, D419, D420, D421, D429, D430, D431, D432, D433, D434, D437, D439, D440, D441, D442, D443, D444, D445, D446, D447, D448, D449, D45X, D460, D461, D462, D463, D464, D467, D469, D470, D471, D472, D473, D477, D479, D480, D481, D482, D483, D484, D485, D486, D487, D489, N600, N601, N602, N603 | ICD-10 |
| Coronary heart disease | G3...-G309. , G30B.-G330z (excluding G310.) , G33z.- G3401 , G342.-G35X. , G38..- G3z.. , Gyu3.% (excluding Gyu31) | Read |
|  | I200, I201, I208, I209, I210, I211, I212, I213, I214, I219, I220, I221, I228, I229, I230, I231, I232, I233, I234, I235, I236, I238, I240, I241, I248, I249, I250, I251, I252, I253, I254, I255, I256, I258, I259, | ICD-10 |
| Heart failure | G58..% , G1yz1 , 662f.-662i | Read |
|  | I500, I501 ,I509,I110,I130 | ICD-10 |
| Stroke and transient ischaemic attack | G61..% (excluding G617.) , G63y0-G63y1 , G64..% , G66..% (excluding G669.) , G6760 , G6W.. , G6X.. , Gyu62-Gyu66 , Gyu6F , Gyu6G | Read |
|  | G45, I63, I64 | ICD-10 |
| Chronic obstructive pulmonary disease | H3..., H30.., H300., H301., H302., H30z., H31.., H310., H3100, H310z, H311., H3110, H3111, H311z, H312., H3120, H3121, H3123, H312z, H313., H31y., H31y1, H31yz, H31z., H32.., H320., H3200, H3201, H3202, H3203, H320z, H321., H322., H32y., H32y0, H32y1, H32y2, H32yz, H32z., H34.., H340., H341., H34z., H35.., H350., H351., H352., H3520, H3521, H352z, H353., H354., H355., H356., H357., H35y., H35y0, H35y1, H35y2, H35y3, H35y4, H35y5, H35y6, H35y7, H35y8, H35yz, H35z., H35z0, H35z1, H35zz, H36.., H37.., H38.., H39.., H3A.., H3B.., H3y.., H3z.., H4640, H4641, H5832, Hyu30, Hyu31, 173J., 173K., 173L. | Read |
|  | E100, E101, E102, E103, E104, E105, E106, E107, E108, E109, E110, E111, E112, E113, E114, E115, E116, E117, E118, E119, E120, E121, E122, E123, E124, E125, E126, E127, E128, E129, E130, E131, E132, E133, E134, E135, E136, E137, E138, E139, E140, E141, E142, E143, E144, E145, E146, E147, E148, E149 | ICD-10 |
