# Supplementary material for: Cardiac interventions in Wales: A comparison of benefits between NHS Wales specialties
Source: PLoS One. 2024 Feb 9;19(2):e0297049. doi: 10.1371/journal.pone.0297049 (PMC10857708; doi:10.1371/journal.pone.0297049)
Supplement: Attachment — Submitted filename: Letter_of_rebuttal.docx [file pone.0297049.s005.docx]

26th October 2023

Dear Amirmohammad Khalaji,

Re: PONE-D-23-25402

Cardiac interventions in Wales: A comparison of benefits between NHS Wales specialties

Thank you for your email dated 11th September 2023 inviting us to revise our paper. We are very grateful for the expert feedback, which we have carefully considered and addressed.

We detail the comments needing action in bold type, and our responses in italics as follows:

**Please ensure that your manuscript meets PLOS ONE's style requirements, including those for file naming…**

*Manuscript style format has been reviewed and actioned where needed.*

**We note that the grant information you provided in the ‘Funding Information’ and ‘Financial Disclosure’ sections do not match.**

*The ‘Funding Information’ and ‘Financial Disclosure’ sections both state WHSSC as the funding source.*

**Please include captions for your Supporting Information files at the end of your manuscript, and update any in-text citations to match accordingly.**

*Captions for Supporting Information files have been added to the end of the manuscript.*

**Have the authors made all data underlying the findings in their manuscript fully available?**

**…**

**Reviewer #2: No**

*The raw data used in this study was accessed via the SAIL Databank, extraction and onward sharing of the data is prohibited by condition of use, however, any accredited researcher can apply to access SAIL. A paragraph detailing this has been added to the manuscript, entitled “Data”, immediately before the references.*

**Is the manuscript presented in an intelligible fashion and written in standard English?**

**…**

**Reviewer #1: No**

*Adjustments to improve the readability of the manuscript have been made throughout the text.*

**Reviewer #1: Davies et al. have performed a study on the assessment of specialized healthcare service interventions in Wales. There are some points that need to be considered.**

**MAJOR Remarks:**

**- The English language of the manuscript can be improved; for instance, in lines 33 and 34. Significant changes should be made all over the manuscript.**

*Lines 33 and 34 have been re-written, and adjustments to improve the readability of the manuscript have been made throughout the text.*

**- The rationale and aim of this study should be clearly described at the end of the introduction.**

*Study aims have been added to the end of the introduction section.*

**- The first paragraph of the discussion should highlight the main findings of the study summarized.**

*Discussion of results in results section has been moved into beginning of discussion section*

**- Discussion should focus on the comparison of these findings with similar studies.**

*References [29-31] to other studies have been added to the discussion section.*

**MINOR Remarks:**

**- In the abstract, what do the authors mean by comorbidity and deprivation?**

*Description of comorbidity as “Charlson” and deprivation as “Welsh Index of Mass Deprivation” have been added into the abstract. References for these occur in the methods section ([27] and [25] respectively).*

**Reviewer #2: In this study, authors have assessed the cost-effectiveness of cardiac interventions and identified the effect of different factors and disparities on the overall treatment cost using comprehensive electronic health record data.**

**This study addresses the critical issue of equitable access to specialized healthcare services, provides evidence-based insights, and has the potential to influence healthcare policy and resource allocation. It contributes valuable information to the ongoing efforts to improve healthcare delivery and ensure that all population members have fair and equal access to needed medical interventions.**

**Nevertheless, there are several points that can improve the quality of the manuscript.**

**1- The introduction could benefit from a more concise statement of the research question or hypothesis the study aims to address at the end of this section.**

*Study aims have been added to the end of the introduction section.*

**2- In the methods section, the authors must discuss in more detail how the missing data were addressed (patients with follow-ups or interventions in centers outside the current HER database ) and if any sensitivity analyses were performed.**

*Further text to address the issue of missing data has been added to the methods section, under the “data extraction” sub-heading.*

**3- The authors need to explain how the covariates were chosen for the adjusted analysis .**

*Additional text to inform covariate selection has been added at the beginning of the “covariates” sub-section in the Method section.*

**4- It is better to perform a survival analysis and present KM curves for survival during the follow-up time. If this is not possible, the limitations section should mention the reason .**

*We are grateful for this suggestion, but do not consider survival analysis appropriate for our aims: Survival analysis / risk of time to an event was not the main objective for the analysis, rather, the study aimed to provide measures of cost of health resource use across different services. Mortality was used simply to adjust costs by observed follow up time for each patient and reported as a descriptive statistic rather than an indication of survival probability following an intervention.*

**5- In the discussion section, the authors need to discuss possible reasons behind the study findings. For example. The reason behind the observed association between deprivation and costs.**

*Discussion of the complexity between the association between deprivation and increased health resource has been added to the discussion section.*

**6- I suggest that authors compare their results to previous studies in the discussion section (Survival rates, elective/emergency ratios, Costs, ...)**

*References [29-31] to other studies have been added to the discussion section. With regard to survival analysis, our aim was not to quantify risk of an event, but rather cost/measure of health resource use across settings.*

**7- The authors should briefly explain how early investment in the pathways can reduce overall costs and, if possible, add some suggestions on improving cost-effectiveness according to the study results.**

*Further text has been added to the discussion section, relating to the benefits of early investment in the patient pathway.*

**8- Some sentences in the introduction are quite long and complex, which may hinder readability. Consider breaking down some of these sentences into smaller, more digestible portions for improved clarity.**

*General text simplification has been applied throughout the revised manuscript to try and improve readability*

**9- Please proofread the manuscript regarding Punctuation, consistency in Hyphen Usage, and Use of acronyms.**

*The revised manuscript has been proof read regarding these points.*

Yours Sincerely,

Gareth Davies (Swansea University), on behalf of the co-authors
