# Supplementary material for: Cardiac interventions in Wales: A comparison of benefits between NHS Wales specialties
Source: PLoS One. 2024 Feb 9;19(2):e0297049. doi: 10.1371/journal.pone.0297049 (PMC10857708; doi:10.1371/journal.pone.0297049)
Supplement: Attachment — Submitted filename: 2nd_letter_of_rebuttal.docx [file pone.0297049.s006.docx]

12th December 2023

Dear Amirmohammad Khalaji,

Re: PONE-D-23-25402R1

Cardiac interventions in Wales: A comparison of benefits between NHS Wales specialties

Thank you for your email dated 14th November 2023 inviting us to modify the first revision of our paper. Again we are very grateful for the expert feedback.

We detail the comments needing action in bold type, and our responses in italics as follows:

**Reviewer #2: I thank the authors for adequately addressing the comments. I believe the manuscript looks much better now. I only have two remaining comments:**

**- In response to reviewer comments, the authors have moved almost all of the results section to the beginning of the discussion. I suggest these parts remain in the results section and only a summary and the highlights of these results be added to the beginning of the discussion in one short paragraph.**

*The results at the start of the discussion have been moved back into the results section, and a more concise paragraph summarising the main findings has been added to the beginning of the discussion section.*

**- Additional fluency modifications are required, especially in lines 162-165.**

*Lines 162-165 have been re-written to improve readability.*

Yours Sincerely,

Gareth Davies (Swansea University), on behalf of the co-authors
